# A comprehensive review and benchmark of differential analysis tools for Hi-C data

**DOI:** 10.1093/bib/bbaf074

**Published:** 2025-03-04

**Authors:** Elise Jorge, Sylvain Foissac, Pierre Neuvial, Matthias Zytnicki, Nathalie Vialaneix

**Affiliations:** GenPhySE, Université de Toulouse, INRAE, ENVT, 31326 Castanet-Tolosan, France; GenPhySE, Université de Toulouse, INRAE, ENVT, 31326 Castanet-Tolosan, France; Institut de Mathématiques de Toulouse, UMR 5219, Université de Toulouse, CNRS UPS, 31062 Toulouse, France; Université Fédérale de Toulouse, INRAE, MIAT, 31326 Castanet-Tolosan, France; Université Fédérale de Toulouse, INRAE, MIAT, 31326 Castanet-Tolosan, France

**Keywords:** Hi-C, differential analysis, statistical tests, benchmark

## Abstract

**Motivation:**

The 3D organization of the genome plays a crucial role in various biological processes. Hi-C technology is widely used to investigate chromosome structures by quantifying 3D proximity between genomic regions. While numerous computational tools exist for detecting differences in Hi-C data between conditions, a comprehensive review and benchmark comparing their effectiveness is lacking.

**Results:**

This study offers a comprehensive review and benchmark of 10 generic tools for differential analysis of Hi-C matrices at the interaction count level. The benchmark assesses the statistical methods, usability, and performance (in terms of precision and power) of these tools, using both real and simulated Hi-C data. Results reveal a striking variability in performance among the tools, highlighting the substantial impact of preprocessing filters and the difficulty all tools encounter in effectively controlling the false discovery rate across varying resolutions and chromosome sizes.

**Availability:**

The complete benchmark is available at https://forgemia.inra.fr/scales/replication-chrocodiff using processed data deposited at https://doi.org/10.57745/LR0W9R.

**Contact:**

nathalie.vialaneix@inrae.fr

## Introduction

Chromosomes are highly compacted within the cell nucleus, resulting in the spatial proximity of linearly distant genomic positions [1]. Hi-C [2] is a widely used technology to profile the 3D organization of the genome. It does so by estimating the spatial proximity between pairs of genomic positions through their frequency of interaction. The typical output of a Hi-C experiment, after preliminary data preprocessing, is usually summarized as a symmetric matrix of counts, where the entry $(i,j)$ (or $(j,i)$) corresponds to the number of interactions registered during the Hi-C experiment between genomic regions (“bins”) $i$ and $j$. Hi-C has been widely used to uncover structural genomic elements at different hierarchical levels, such as A/B chromatin compartments, TADs, and loops [1–3]. Many computational tools exist to call these structures from Hi-C data, with variable reliability however [4–6].

Changes in 3D structures have been implicated in gene expression, cell division, cell differentiation, developmental disorders, and cancers [7–9]. This underscores the need for reliable methods and tools to compare Hi-C data across different conditions. One approach to comparing Hi-C data is to compute a similarity score for a pair of matrices, either at the level of the entire matrix (matrix-level) or for specific genomic regions (bin-level). Gunsalus et al., 2023 [10] reviewed several methods for the pairwise comparison of Hi-C matrices, classifying them into three categories: *basic methods*, which directly compute a similarity score (e.g. a correlation) between two matrices [11], *map-informed methods*, which first calculate a Hi-C-related metric along a 1D track for each matrix separately (e.g. directionality index) and then compare the resulting tracks [12], and *feature-informed methods*, which predict specific chromatin structures for each matrix (e.g. TAD boundaries or chromatin loops) before comparing the predictions [13]. While these methods offer various similarity or dissimilarity metrics, none provide statistical guarantees such as $p$-values. Moreover, they focus solely on pairwise matrix comparisons without incorporating biological replicates.

Another approach to comparing Hi-C data is differential analysis. Instead of quantifying the overall similarity between two Hi-C matrices (one for each condition), differential analysis aims at identifying local differences with statistical guarantees, often leveraging biological replicates for each condition. Following the previous classification, some of the methods can be considered as *map-informed*, as they use 1D metrics to detect differential structures such as TAD boundaries [14, 15] or chromatin compartments [16] at the bin level. Other methods fall under the *feature-informed* category, aiming to identify differential TADs, for instance [17]. However, most tools for differential analysis of Hi-C data do not fit into these categories, as they test for differences at the level of the bin pair, focusing on interaction counts between genomic regions [18–25]. To our knowledge, these methods have not been extensively reviewed or benchmarked. A recent book chapter [26] describes a few (four) such methods. While it provides technical instructions on their use and visualization of results, it does not evaluate the quality of the results.

To address this gap, we propose a comprehensive review of the following tools for the differential analysis of Hi-C data: **ACCOST** [18], **CHESS** [27], **diffHic** [19], **FIND** [20], **HiCcompare** [21], **HiCDCPlus** [22], **multiHiCcompare** [23], **Selfish** [24], and **sslHiC** [25]. We also considered a former version of **HOMER** (The version available and documented at http://homer.ucsd.edu/homer/interactions/.), which included a test to perform comparisons between two matrices, although 3D structure changes are not the primary focus of that tool. Our review provides a detailed technical description of each tool, focusing on implementation aspects, usability, and scalability. We explain the differences between the statistical methods employed by these tools and analyze their expected impacts on the results.

We also conducted two extensive benchmarks of the tools using real Hi-C data from the literature. The first benchmark used Hi-C data generated from a human tissue sample, with an artificially introduced ground truth to allow a quantitative evaluation of each tool’s precision and power. The second benchmark involved Hi-C data from a CTCF depletion study during mouse cell cycle progression, evaluating the biological relevance of each tool’s results by comparing them to findings from ChIP-seq experiments.

The article is organized as follows: The second section “Methods” reviews the statistical grounds of the different tools in a rigorous way. The third section “Implementation and usability” describes the technical aspects of the tools. The fourth section “Numerical experiments” introduces our benchmark protocol and the fifth section “Results” analyses the tools’ performances.

## Methods

### Methodological overview of the tools

This article covers tools that all aim at answering the same question: given a set of $n$ Hi-C matrices, $\mathcal{M}_{1}$, …, $\mathcal{M}_{n}$, belonging to $K$ different groups of biological interest (that we will call “conditions”), are we able to find bin pairs with significantly different interaction counts between conditions? While several descriptive metrics (such as correlation or other similarity measures) can be used for this purpose, we focus on approaches that provide statistical guarantees for identified bin pairs. Such approaches perform one statistical test for each bin pair. The result of each of these tests can be summarized by a $p$-value (or an adjusted $p$-value), which quantifies the statistical evidence of a significant difference.

**Figure 1:**
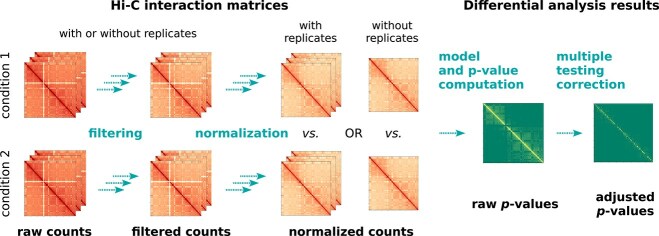
A schematic representation of the typical workflow for differential analysis of Hi-C matrices at the bin-pair (pixel) level. Input matrices from two conditions, with or without replicates, are first filtered and normalized (first two steps). Statistical tests are then conducted to generate raw $p$-values, which are subsequently adjusted for multiple testing correction (last two steps). Additionally, a (log) fold change matrix can be generated, representing the ratio of average interaction values between conditions for each bin pair (not shown).

The tools discussed in this article all have a common workflow (Fig. 1). In short, this workflow takes Hi-C matrices from different conditions and performs a statistical test, which results in a $p$-value (or an adjusted $p$-value) for each bin pair. **CHESS** is the only tool that slightly differs from this description because it provides $p$-values for fixed-sized windows of the Hi-C matrix (and not for every bin pair; see Section “Methodological background of the tools”).

As shown in Fig. 1, the Hi-C differential analysis workflow can be decomposed into four main steps:

- *filtering*, which consists in removing bin pairs considered not relevant from the analysis in all Hi-C matrices;
- *normalization*, which consists in making bin pairs in a matrix or bin pairs between different matrices more comparable;
- *model and $p$-value computation*, which is the core of the statistical analysis and performs a test on all remaining bin pairs, using normalized interaction values;
- *multiple testing correction*, which aims at accounting for the fact that a large number of tests have been performed.

Most tools operate at the chromosome level, detecting intra-chromosomal (*cis*) differential interactions only. However, **diffHic**, **HOMER**, and **sslHiC** can also detect inter-chromosomal (*trans*) interactions.

The steps of the Hi-C differential analysis workflow and the various options used by different tools are described in detail in the sections below. Table 1 summarizes the main methodological characteristics of the tested tools in relation to the steps of this workflow.

**Table 1:** Methodological description of the tools (summarized; for further details see the text)

	Preprocessing		Model					p-values	
Tool	Filtering	Normalization	Data distribution assumption	Leverages replicates	2D-aware approach	Allows covariates or $ K > 2$ conditions	Type of model/test	Provides raw $p$-values	$p$ -value correction
**ACCOST**	Bin quality	(ICE)	NB	✓	$\times $	$\times $	exact test	✓	(BH)
**CHESS**	Bin quality	(ICE)	None	$\times $	✓	$\times $	SSIM	✓	$\times $
**diffHic**	Low counts	MA, KR	NB	✓	$\times $	✓	GLM	✓	BH
**FIND**	$\times $	(MA), KR	SPP	✓	✓	$\times $	exact test	$\times $	BH by distance
**HiCcompare**	User-defined	TSS, MD	None	$\times $	$\times $	$\times $	$Z$ -score	✓	BH by distance
**HiCDCPlus**	Low counts, bin quality	(ICE)	NB	✓	$\times $	$\times $	exact test	✓	BH
**HOMER**	$\times $	ICE	NB	✓	$\times $	$\times $	GLM	✓	BH
**multiHiCcompare**	Low counts	MD	NB	✓	$\times $	✓	GLM	✓	BH by distance
**Selfish**	Low counts, distance	KR	None	$\times $	✓	$\times $	$Z$ -score	$\times $	BH
**sslHiC**	$\times $	TSS, min/max	None	$\times $	✓	$\times $	GNN	$\times $	BH

ICE: iterative correction and eigenvector decomposition; KR: Knight–Ruiz; NB: negative binomial; SPP: spatial Poisson process; SSIM: structured similarity index; GLM: generalized linear model; GNN: graph neural network; BH: Benjamini–Hochberg. Items between parentheses correspond to steps advised by the method authors but not implemented in their tool

### Filtering

Several tools propose to remove some bin pairs before the analysis. The rationale behind this step is that low quality bin pairs or bin pairs with low interaction counts have little (if any) chance to be identified as differential but increase the number of hypothesis tests that are performed. Including such bin pairs can affect the test power, due to stronger multiple testing correction (see corresponding section below for further details). Discarding bin pairs before the test (and independently from its result) is a standard way to reduce this impact [28, 29].

The most common filters used in Hi-C differential analyses are:

- *low count filtering* (implemented in **diffHic**, **HiCcompare**, **HiCDCPlus**, **multiHiCcompare**, and **Selfish**). These filters simply remove, from the analysis, bin pairs that have interaction counts below a certain threshold. This threshold is either user-defined (i.e. all bin pairs for which the average or total interaction counts across analyzed matrices is below the threshold as in **diffHic**, **HiCcompare**, **multiHiCcompare**, and **Selfish**) or data-driven (the threshold is obtained as an estimation of a “background signal” from the data as in **diffHic** and **HiCDCPlus**);
- *bin quality filtering* (implemented in **CHESS**, **ACCOST**, and **HiCDCPlus**). These filters remove bin pairs including at least one bin with low mappability or, alternatively, bin pairs for which the interaction counts are below the expected (data-driven) value considering the bin mappability and GC content. These filters require that mappability or GC content is provided for each bin.

Additionally, **Selfish** allows to discard all bin pairs for which the genomic distance between the two bins is larger than a certain (user-defined) value, typically targeting bins with low interaction counts. Also, **HiCcompare** has an option to let users specify a set of bins that should not be considered in the analysis. Supplementary Table S1 summarizes the type of filters available in each tool.

### Normalization

Hi-C matrix normalization is an important step of the workflow. It aims at removing technical or biological biases that can impede a fair comparison between bins or between matrices [30]. The main known biases that can affect Hi-C data analysis are scale differences between matrices (e.g. due to differences in sequencing depths), scale differences between bins in a given matrix (e.g. due to differences in mappability), or effects of the genomic distance in interaction counts within a given matrix. In the current section, we discuss these biases, their impact on the analysis, and how the different tools address them.

**Differences in total interaction counts between matrices.** Total interaction counts across all bin pairs can differ between matrices due to experimental factors, such as variations in sequencing depth or library complexity. To prevent false positive predictions that would incorrectly label bin pairs as “differential”, these technical artifacts must be accounted for, as commonly done in RNA-seq [31] or ChIP-seq data [32, 33] differential analyses.

The most straightforward method to correct this bias is the total sum scaling (TSS) that simply aligns the total counts of all matrices in the dataset (implemented in **sslHiC** and advised, but not implemented, in **HiCcompare**). However, this approach has been shown to be generally inefficient for sequencing data, as it is strongly influenced by large outlier counts [31].

Hence, the article of Lun & Smyth, 2016 [33] emphasizes the need for an adequate between-matrix normalization and proposes the MA correction (correction of the trend in an MA plot, where the difference “M” between two or more matrices is displayed as a function of their average count “A”). This correction, performed by cyclic locally estimated scatterplot smoothing (LOESS), has been shown to be efficient for ChIP-seq data and robust to a large proportion of low counts. It is implemented in **diffHic** (and advised in **FIND** but not implemented).

A more sophisticated alternative is used in **HiCcompare** and **multiHiCcompare**. The MA correction is replaced by an MD correction (where D stands for the genomic distance between bin pairs, instead of its average count). However, since A and D are strongly related in Hi-C matrices (the larger the distance between the two bins of a pair, the lower the count for this bin pair), both methods are expected to result in similar corrections.

Finally, although this does not strictly aim at correcting differences in sequencing depths, **sslHiC** also implements a min/max normalization applied to $\log _{10}$-transformed matrices so as to make all counts in a given matrix lie between 0 and 1 (and thus be more comparable across matrices).

**Differences in total counts between bins within a given matrix.** The total number of interactions assigned to a specific bin or over a given genomic region depends on local properties of the genomic sequence, such as GC content, mappability, or restriction site density [34]. Since the purpose of differential analysis is to compare bin pairs between matrices and not bin pairs within the same matrices, correcting for these biases is not strictly necessary from a statistical perspective. However, several tools nevertheless recommend or implement methods for correcting these biases.

Among the most popular methods for within-matrix normalization, non-parametric methods do not explicitly use GC content or mappability values to remove biases between bin counts. On the contrary, similarly to TSS normalization, they align the observed total count across all bins of a given matrix. These include iterative correction and eigenvector decomposition (ICE) [35], implemented in **HOMER**, or Knight–Ruiz (KR) matrix balancing [36], implemented in **diffHic**. Other tools (**FIND** and **Selfish**) benefit from the juicer data format [37] and embed values that allow for KR correction. Finally, **CHESS** and **HiCDCPlus** recommend the ICE correction but do not implement it.

An alternative to choosing a specific method to correct between-bin biases is to let users provide bin-specific correction values. This is the course of action taken by **ACCOST** (that recommends using ICE but allows for any other type of bin correction values to be used).

Finally, note that if all bin sums in a given matrix are aligned to the same total count $B$ (e.g. $B=1$ for KR correction), then it is sufficient to use the same $B$ for all matrices to align, at the same time, overall total interaction counts between matrices.

**Genomic distance related differences between interaction counts within a given matrix.** Hi-C matrices are strongly structured with respect to the genomic distance between bin pairs. Likewise biases between bin total counts within a given matrix, this bias does not necessarily require correction but is nevertheless accounted for in several tools.

One of the most popular approach to correct for this bias is to compute an “observed over expected” matrix. The interaction count of a bin pair is divided by the average interaction counts of all bin pairs with the same genomic distance. This approach is implemented in **CHESS**. Similarly, the interaction count of a bin pair at the same genomic distance can be centered and reduced to unit variance (as implemented in **Selfish**) or scaling factors for bin pairs at the same genomic distance (e.g. median) can be computed and used for normalization (as implemented in **ACCOST** and **HiCDCPlus**).

**Other normalizations.**

Other methods designed to correct various other biases are also implemented: **diffHic** proposes a method based on DNA copy number variation (CNV) estimation to correct for this bias. However, as discussed by Servant et al., 2018 [38], CNV could be of interest in cancer studies and it is therefore not necessarily sound to always use this correction.

Finally, note that all tools that use the genomic distance between bins for the normalization are restricted to detect intra-chromosomal (*cis*) differential interactions only and cannot consider inter-chromosomal (*trans*) interactions.

Supplementary Table S2 summarizes the different normalization options offered by the tools.

### Methodological background of the tools

This section describes the methodological premises and solutions of the different tools. The tools can be classified according to the following two questions:

- Can the tool *use biological replicates* to perform the test (i.e. handle more than one matrix in each condition)? When biological replicates are available, it is still possible to use a tool designed to only compare one matrix in each condition by merging (computing the sum of) the replicates of each condition. However, it is strongly advised, from a statistical perspective, that these replicates are used in order to relate the inter-condition variability to the intra-condition variability.
- Does the tool consider interaction counts as independent from each other, or does it try to take advantage of the fact that two bin pairs, $(i,j)$ and $(i^{\prime},j^{\prime})$ in the matrix, tend to have more similar interaction counts when they are close to each other (e.g. $|i-i^{\prime}| + |j-j^{\prime}|$ is “small”)? We will use the term “2D-agnostic” for the tools that consider bin pairs independent and the term “2D-aware” for the tools that account for this property.

These two typology levels for the tools are provided in the two columns of Table 1 named “use of replicates” and “2D-aware”. We now give a brief overview of each tool based on the answer to these two questions.

#### Comparison of two matrices

The tools designed to perform differential analysis between two matrices are: **HiCcompare** (2D-agnostic), **CHESS**, **Selfish**, and **sslHiC** (2D-aware).

**2D-agnostic method.** A 2D-agnostic method means that a measure of the difference between the two matrices is obtained at bin pair level and transformed into a $Z$ score, from which a $p$-value is derived using the Gaussian distribution. More precisely, **HiCcompare** uses the M-value (log-fold change between the two matrices) of the interaction count to obtain a $Z$ score.

**2D-aware methods.** Existing 2D-aware methods that perform tests between two matrices are based on different premises: **CHESS** first partitions the Hi-C matrix into fixed-size square submatrices and computes a structural similarity index (SSIM). This index is commonly used in imaging analysis to quantify the similarity between two matrices. It depends on the average signal in each submatrix, the variance of the signal in a given submatrix, and the signal correlation between the two submatrices. A $p$-value is then derived for each square from this index, quantifying the exceptionality of the observed index with respect to a background model.

**Selfish** performs a sort of “local smoothing” of the matrices: For each bin pair, it applies Gaussian filters centered at the bin pair, with increasing radius. The idea is to take advantage of the spatial self-similarity in contact maps to improve statistical evidence. Differences of the Gaussian filter evolutions between the two matrices are then assumed to be Gaussian, from which a $p$-value is derived for each radius. The final $p$-value is defined as the minimum radius-specific $p$-value across radii. However, since no multiple testing correction is applied at this stage, the resulting $p$-values are invalid.

Finally, **sslHiC** is based on a graph neural network (GNN) [39, 40]. The idea is to represent a Hi-C matrix as a graph in which bins are nodes and positive interaction counts are edges (weighted by the interaction count). Bin pairs of the form $(i,i+1)$ (linking two bins that are neighbors on the chromatin) are also linked with an edge to encode the genome structure in the Hi-C graph. The authors propose a new architecture of GNN, which they call “edge-enhanced GNN” (EEGNN) that aims at better exploiting the information carried by edges in the message passing process of the GNN. Using this architecture, all the bin pairs $(i,j)$ in the matrix are represented by their embeddings $h^{k}_{(i,j)}$, $d$-dimensional vectors organized in different layers, $k$. The method is fully aware of the whole matrix since the embedding $h^{k}_{(i,j)}$ at layer $k$ is passed to the other bin pairs that share a common node to compute their embeddings at layer $k+1$. The method finally derives a $p$-value for $(i,j)$ by assuming Gaussian distribution of the Euclidean distance between embeddings $h^{K}_{(i,j)}$ of the two matrices in the last layer $K$.

#### Comparison of multiple matrices for each condition.

Tools that leverage replicates to perform differential analysis are **ACCOST**, **diffHic**, **HiCDCPlus**, **HOMER**, **multiHiCcompare** (2D-agnostic), and **FIND** (2D-aware). As explained above, an important advantage of these tools from a statistical perspective is that they can account for the variability across replicates within each condition (e.g. by computing variances, which cannot be done when a single replicate is available).

**2D-agnostic methods.** 2D-agnostic tools that account for replicates (**ACCOST**, **diffHic**, **HiCDCPlus**, **HOMER**, and **multiHiCcompare**) all assume that the interaction counts follow a negative binomial (NB) distribution. This is a standard hypothesis already used in other differential analysis methods for sequencing data, notably for RNA-seq. More precisely, **diffHic** and **multiHiCcompare** integrate **edgeR** [41] functions that fit a NB generalized linear model (GLM) (and thus directly benefit from the flexibility of this framework, able to account for complex experimental designs). The main difference with the standard RNA-seq pipelines is the addition of an offset derived from the MA (**diffHic**) or MD (**multiHiCcompare**) normalization in the NB GLM. Similarly, **HiCDCPlus** and **HOMER** use **DESeq2** [42] and differ from **DESeq2** by the preprocessing performed on Hi-C matrices, especially the filtering step described in the “Filtering” section. Alternatively, **HOMER** can also use an **edgeR** model. Even if it does not directly depend on **DESeq2**, **ACCOST** also derives its method from **DESeq2**’s NB model, plugging the bin-specific correction values described in “Normalization” into the NB GLM method of **DESeq2**.

**2D-aware method.** The only 2D-aware tool able to account for replicates is **FIND**. In **FIND**, a bin pair is described by its position $(i,j)$ in the matrix 2D structure and its interaction counts across matrices. The resulting triplet is distributed as a spatial Poisson process (a count process that has a spatial structure) with condition-specific intensity parameter $\lambda _{1}$ and $\lambda _{2}$. A first-level $p$-value for the test of the null hypothesis $\lambda _{1}=\lambda _{2}$ is then obtained for each bin pair. The final $p$-value at each bin pair is obtained by aggregating the first-level $p$-values in the local neighborhood around the bin pair, using the $r$-ordered $p$-value (rOP) method [43]. However, the resulting $p$-value may not be valid since the rOP method assumes independence between the hypotheses to be aggregated.

### Multiple testing correction

All the evaluated tools perform one statistical test for each bin pair $(i,j)$, with **FIND** and **Selfish** deriving this $p$-value by aggregating results from other previous tests. Therefore, a multiple testing correction is necessary to control false positives–bin pairs identified as differential by chance rather than due to a true difference in interaction levels between the two conditions. Multiple tests in genomic studies are generally handled by controlling the false discovery rate (FDR). The FDR corresponds to the expected proportion of false positives among the bin pairs called significant by a given method. The state-of-the art method for FDR control is the Benjamini–Hochberg (BH) method [44].

However, multiple testing correction is handled in different ways across tools. **diffHic**, **HiCDCPlus**, **HOMER,** and **sslHiC** implement FDR control using the BH method. While **ACCOST** does not directly provide multiple testing correction, its authors also used the BH method in [18]. A different strategy is implemented in **HiCcompare**, **multiHiCcompare**, and **FIND**. These methods perform multiple testing correction on a per-distance basis, also using the BH method. This implies that the FDR of their results is (theoretically) not globally controlled at the chromosome level, which means that more false positives can be expected for these tools. Notably, as the typical use case of the tools considers individual chromosomes, looking for differences in *cis*-interactions, multiple testing correction is not performed by these methods at the genome-level.

### Handling more complex experimental designs

Finally, **diffHic** and **multiHiCcompare** are designed to perform a test between more than $K=2$ conditions or are able to include external covariates in the model. The latter is useful when an experimental factor is not of primary interest for the differential analysis but might influence the results (e.g. a noise effect, like the sex or the tissue, could mask the differences due to the factor of interest, like the treatment). In this case, it is common practice to account for this covariate as a “blocking factor,” correcting the effect without testing for it. However, due to the high cost of Hi-C data generation, such complex designs (involving more than two conditions and/or covariates) remain rare. As a result, while these features may be valuable for future experimental designs, they are not the primary focus at present.

## Implementation and usability of the tools

Table 2 summarizes technical information for each tool, including the programming language, whether the tool is packaged and easy to install, which input formats are handled, whether a documentation is provided, and when it has been last updated.

**Table 2:** Technical description of the tested tools

Tool	Language	Installation	Input format	Documentation	Last modification	Code repository
**ACCOST** [18]	Python2 & R	Python scripts	own tsv	Basic local HTML manual	2022-07	ACCOST $^{1}$
**CHESS** [27]	Python3	pip	.hic/.cool/.fanc	Complete “readthedocs” documentation	2023-07	Github
**diffHic** [19] (v1.26.0)	R	Bioconductor	GInteractions R object	Manual + Bioconductor vignette	2024-03	Github
**FIND** [20] (v1.0.0)	R	source R package	HiC-PRO	Basic online manual + local vignette	2022-11	Bitbucket
**HiCcompare** [21]	R	Bioconductor	HiC-PRO/own tsv	Manual + Bioconductor vignette	2023-06	Github
(v1.16.0)						
**HiCDCPlus** [22]	R	Bioconductor	GInteractions R object	Manual + Bioconductor vignette	2022-06	Github
(v1.2.1)						
**HOMER** [51]	Perl & C++	Perl & C++ script	FASTQ reads	Complete website	(not mentioned)	none
**multiHiCcompare** [23]	R	Bioconductor	HiC-PRO/own tsv	Manual + Bioconductor vignette	2022-04	Github
(v1.12.0)						
**Selfish** [24]	Python3	pip/container	.hic/.cool/HiC-PRO/own tsv	Basic manual	2021-05	Github
**sslHiC** [25]	Python3	Python scripts + conda env	.cool/.mtx/.npy/.npz	Basic online manual	2023-04	Github

Dates of last modification were collected in November 2023. Version of the tool used in the benchmark (when provided by the tool) is indicated in the first column. $^{(1)}$ We found three repositories for **ACCOST** code, one on Bitbucket and two on Github (one of them has not been updated for 8 years). We contacted the authors who indicated us the repository to consider

### Inputs and input formats

Almost all the tested tools assume that the raw sequencing reads have preliminary been processed with a Hi-C data analysis pipeline and consequently converted into interaction matrices. Notable exceptions are **diffHic**, which can also handle BAM or FASTQ files, and **HOMER**, which requires BAM or FASTQ files.

During the construction of the interaction matrix, paired-end reads are usually mapped to a genomic reference sequence. Chromosomes are then discretized into fixed-size bins, and interaction matrices are obtained by counting for each bin pair the number of read pairs that link the corresponding bins. In short, interaction matrices are essentially symmetric square matrices with non-negative entries and many zeros.

Several file formats have been proposed to store these matrices, with different degrees of adoption. Although none of them has become the universal standard yet, a few are used by several tools. Such common formats include binary (and possibly compressed) formats, like the .hic [37], .cool, .mcool [45] and .fanc [46] formats, and text-based formats like the HiC-PRO [47] or BEDPE [48] formats. A majority of the tested tools (namely **CHESS**, **FIND**, **HiCcompare**, **multiHiCcompare**, **Selfish**, and **sslHiC**) use these standard formats (Table 2). Note that **sslHiC** can take as input a .cool file or a contact matrix file similar to the one generated by **HiC-PRO**. In the latter case, unlike the other tools, it does not require an index file but only the matrix resolution (only certain resolutions are allowed; see Methods). The matrix file is then given as an .mtx file in the Matrix Market format, or can directly be passed as a binary Python/**numpy** file (.npy or .npz).

The other tools use more specific formats. For instance, **ACCOST** requires a tab-delimited format file, with columns <chr1> <mid1> <chr2> <mid2> <#reads>, where mid$i$ is the position of the middle of bin $i$ ($i=1,2$), and #reads is the raw interaction count. **HiCDCPlus** and **diffHic** use the GInteractions Bioconductor class [49] as input format. **HOMER** is the only tool in the list that exclusively accepts raw reads as input, rather than interaction matrices. As a result, users must map the data with **HOMER**, making it incompatible with pre-existing matrices for differential analysis.

Furthermore, some tools require additional data with the interaction counts. **ACCOST** requires a bin-specific normalization score for each bin, which can be obtained with the ICE method [35], as implemented, e.g. in Bioconductor/**HiC-PRO** package [47] or in **Cooler** [45]. Note that **ACCOST** can accommodate any possible bin-specific bias as input, allowing to use parametric methods based on GC content, mappability, or restriction site density, as long as they provide a score for each bin. Additionally, **ACCOST** requires a mappability score for each bin, but this information is only used to discard some bins from the analysis.

Likewise, **HiCDCPlus** requires GC content information, but the tool can compute it internally as long as the corresponding genome is available from Bioconductor [50]. Optionally, mappability information can also be provided. In contrast to other tools, **CHESS** performs a test and derives a $p$-value only if a set of background regions, where no difference is expected between the two matrices, is provided. Otherwise, **CHESS** only computes similarity scores between the matrices and does not return a $p$-value.

Finally, some of the tools contain format converters, like **HiCcompare** and **multiHiCcompare** that provide functions to convert .hic and .cool files to their own internal format.

Of note, **sslHiC** is the only tool that restricts the bin size. Namely, it can only analyze Hi-C matrices at resolutions 10, 50, or 500 kb, because the authors trained their deep-learning models for these resolutions only.

### Programming languages and packaging

Most of the tools reviewed are implemented in Python and/or R (Table 2), with the exception of **HOMER**. From a user perspective, availability through a package management system (like **pip**, **conda**, or the CRAN repository) is highly valuable because dependencies are usually handled during the installation process, making it much easier to install compared to non-packaged tools. Bioconductor packaging [50] offers additional stability for several reasons: the code is extensively reviewed before acceptance, every release is tested on the three main operating systems, and extensive documentation is required (including a use case vignette). Python packages often rely on an external documentation website, which can be extensive and detailed (such as the ones hosted on the “Read the Docs” documentation service https://about.readthedocs.com/).

From this point of view, the R/Bioconductor packages **diffHic**, **HiCcompare**, **HiCDCPlus**, and **multiHiCcompare** are easy to install, thanks to the Bioconductor common installation process. **FIND** is also easy to install, even if not included in an official package repository.

Similarly, for Python tools, **CHESS** and **Selfish** are easy to install, thanks to **pip**. In addition, **Selfish** also proposes an installation process via a **Docker** or a **Singularity**/**Apptainer** container, providing further reproducibility and robustness. In contrast, **ACCOST** does not offer a **pip** installation and is just provided as Python scripts. For **ACCOST**, the authors simply mention its dependency with Python 2.7 and R, as well as with **numpy**, **scipy**, and some **scikit-learn** libraries. In contrast, **sslHiC** is easier to use thanks to a provided conda environment setting file. **HOMER** includes a script, which installs and configures the tool. A description of the documentation of the tools, together with their comprehensivenesses and readabilites, and a description of issues encountered during installation of the tools are provided as Supplementary Sections 3.1 and 3.2, respectively.

### Illustrative datasets

In addition, having some data included in the tool for illustration is usually appreciated by users. From this perspective, **HOMER** and **Selfish** do not include any dataset. **diffHic** includes a small BAM file used in its manual, while its vignette features three external datasets also mentioned in their article. **FIND**, **HiCcompare**, **HiCDCPlus**, and **sslHiC** include (part of) the processed data from [3] (GEO: GSE12878), which they used in their documentation and also (except for **FIND**) in the results of the article. **ACCOST** also includes part of the same dataset but does not use it in the HTML manual for illustration (this dataset is discussed in their article, however). **sslHiC** also includes datasets simulated from chromosome $21$ of a GM12878 cell line dataset (the original dataset is only used to illustrate another feature of the tool on a replication measure). The simulated dataset consists in three couples of matrices including a certain percentage of simulated differential interactions with varying fold changes ($2$, $4$, and $6$). **CHESS** includes the processed data from [52] (ArrayExpress: E-MTAB-5875) and **multiHiCcompare** part of the data from [53] (GEO: GSE104888). Both use these datasets in their documentation and article.

Note that all datasets are not provided under the same format. **ACCOST** provides compressed .tsv files that correspond to their input format, **CHESS** and **HiCDCPlus** provide .hic files, **FIND** and **HiCcompare** embed data in their tool (they can then be loaded using the function data, directly properly formatted for usage in their functions or as GInteractions objects [49]).

## Numerical experiments

In this section, we present the extensive numerical experiments that we performed to assess the statistical performance of the tools. In particular, we describe the datasets, the tools, and how we designed the tests to evaluate the Type-I error control, the power, and the biological relevance of the results.

### Tested tools

Among the tools described in “Methods,” we excluded three tools from the simulation study:

1. **CHESS** because it is made to provide $p$-values for fixed-sized windows of the Hi-C matrix that “cannot be smaller than 20$\times $ the bin size of the data” (User documentation even recommends to use regions spanning at least 100$\times $ the bin size of the data.), which is hardly comparable with the other tools (that obtain results at a bin pair resolution);
2. **ACCOST** because it is not actively maintained anymore (it resulted in errors with Python. We contacted the authors about this problem which they intend to solve);
3. **HOMER**, which led to an error with our data. We contacted the authors about this problem without success.

Supplementary Table S3 provides the link to the source code and the version or date at which it was accessed for installation.

All tools were launched successfully for all experiments except for:

- **multiHiCcompare** that filtered out all bin pairs in chr 21 experiments with the semi-simulated dataset. All bin pairs were also filtered for chr 13, 14, and 15 of the CTCF depletion dataset. The tool was successful but no results were produced;
- **sslHiC** that we could run only on 500 kb resolution matrices. The tool was successful for this setting but not designed for the other settings.

### Tool parameters

These tools were tested with their default parameters whenever possible. The exceptions to this choice are listed in Supplementary Table S4 and correspond to parameters with no default but required by the tool to work, as for **FIND**.

In addition, by default **FIND** filters out results for which the adjusted $p$-value was above a certain threshold. We turned this filter using qvalue = 1 to retrieve all results (Using this setting results in **FIND** returning adjusted $p$-values equal to one as zeros, which is not desirable. We manually corrected this setting in our code.). We also used the option to split the computation into several chunks (otherwise, using the tool resulted in memory overload).

Similarly, **diffHic** provides several functions that can perform different types of filtering before the differential analysis. In our experiments, we did not filter out bin pairs with a low $\log $CPM but we used their filterTrended filter.

For a given experiment and tool, $p$-values were adjusted independently for each chromosome. The BH procedure [44] was used to adjust $p$-values when the tool did not provide adjusted $p$-values. For tools that perform a per-distance-basis FDR correction (**HiCcompare** and **multiHiCcompare**), we kept their adjusted $p$-values and also computed adjusted $p$-values at the chromosome level (“standard” BH procedure). In the Results section, these two types of results are identified by **HiCcompare** (original adjusted $p$-value of the tool) and **HiCcompare-realFDR** (re-computed adjusted $p$-value). We were unable to perform this correction for **Selfish**, **FIND**, and **sslHiC**, which, unfortunately, do not provide raw $p$-values.

### Semi-simulated data study

We first used a use case where the ground truth of difference locations is precisely controlled. One possibility to do this would have been to rely on a simulation study, generating data from a specific probability distribution. A natural choice for this distribution would be the NB model, since a number of differential analysis tools rely on this distribution (**diffHic**, **HiCDCPlus**, **multiHiCcompare**, and **ACCOST**). However, the evaluation process would have then been biased in favor of these tools. More generally, any choice of a particular distribution induces biases since the true data generating distribution is unknown.

**Figure 2:**
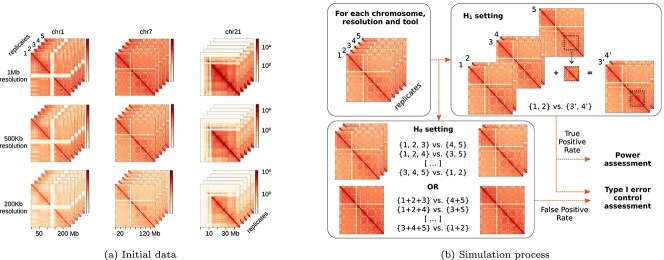
**Design of the simulations.** (a) The available matrices used for the numerical experiments consist in five technical replicates from three chromosomes (1, 7, and 21) generated at three different resolutions (200 kb, 500 kb, 1 Mb). Numbers at the bottom row correspond to genomic positions (in Mb), indicating the size of these matrices. (b) Illustration of the simulation process for Type-I error assessment (H$_{1}$ setting, top right) and assessment of false positive rate (FPR) and true positive rate (TPR) (H$_{0}$ setting, bottom left). Type-I error control was assessed by splitting technical replicates randomly into two groups, while FPR and TPR were assessed by generating artificial true positive examples where read counts are increased in a target zone by adding resampled Hi-C data from the remaining technical replicate.

We therefore used semi-simulated data coming from real Hi-C data. This type of approach has previously been applied multiple times to benchmark tools for, e.g. RNA-seq data [54–56]. More specifically, we used an ENCODE dataset [57] from a Hi-C experiment performed on a human colon sample (experiment accession: ENCSR295BDK), that includes five technical replicates (sequencing runs). To obtain Hi-C matrices, raw sequencing reads of each technical replicate were processed using the nf-core/hic pipeline [58] v1.2.2 on the assembly version GRCh38 of the human genome (see Supplementary Section 4.1 for further details). Hi-C matrices at three different resolutions and for three different chromosomes were finally used, as shown in Fig. 2a. Processed data are available at https://doi.org/10.57745/LR0W9R.

To assess the Type-I error control, we ran each tool on technical replicates randomly split into two groups, where no signal is expected. We also assessed the statistical power of the tests by creating a controlled difference in a given part of some matrices. Figure 2 illustrates (a) the data matrices used and (b) the test protocol.

#### Type-I error control (H$_{0}$ setting)

The quality of statistical tests is usually assessed via their mathematical validity (proper control of the Type-I error, or false discoveries) and by their performance (statistical power or ability to detect true positives). In this first simulation setting, we generated data under the null hypothesis ($\mathrm{H}_{0}$) in which no signal is expected, as described in Fig. 2b.

For each chromosome, we assigned the five technical replicates to two conditions (three replicates in a condition and the other two in the other condition) and processed them with the six tools to extract $p$-values for differential interactions between the two conditions. The $C_{5}^{3} = 10$ possible assignments of the replicates into two groups were obtained and considered as independent experiments (i.e. $p$-values were adjusted independently in each assignment and chromosome).

For tools designed to compare only two matrices (one for each condition), i.e. **HiCcompare** and **Selfish**, we merged the replicates of the same condition into a single matrix before processing the two resulting matrices with the tool (Fig. 2b). Also, **Selfish** results were not symmetric (the $p$-value assigned to the bin pair $(i,j)$ was not always equal to the $p$-value assigned to the pair $(j,i)$ whereas the Hi-C matrix is symmetric by design and logFC were found identical between the two pairs). For instance, for simulation 6, chromosome 21, and resolution 1 Mb, **Selfish** returned a $p$-value of $0.9$ for the pair $(2810, 2805)$ and a $p$-value of $7.4e^{-4}$ for the pair $(2805, 2810)$, as documented in our code repository). To address this, we arbitrarily kept one of the two $p$-values returned by the tool (the one corresponding to $i < j$).

The total number of performed tests, the percentage of significant results (based on $p$-values and adjusted $p$-values) at different risk levels as well as the empirical cumulative density function (ECDF) were obtained for each tool, chromosome, and resolution. Note that not all tools provided raw $p$-values. **FIND**, **Selfish**, and **sslHiC** only returned $p$-values adjusted for FDR control. For these tools, one can only verify that the average number of tests declared significant (at any target FDR level) is zero for a H$_{0}$ setting.

#### Simulations with ground truth signal (H$_{1}$ setting)

The same dataset was used to generate pseudo-simulated experiments corresponding to the existence of a region with a positive signal, as described in Fig. 2b. More specifically, the five technical replicates of each chromosome were used in the following way:

- two replicates were used as the Hi-C matrices of the first condition;
- two other replicates were modified to be used as the Hi-C matrices of the second condition. We first selected a region, called “target zone”, and increased the counts of the matrices of the second condition in this region by adding the corresponding values from the fifth replicate. The target zone consisted of bin pairs where both bins were located within the 20th to 40th percentile range of chromosome length, with 0% representing the start of the chromosome and 100% representing the end.

This simulation setting was designed to obtain a controlled differential area in the matrix approximately mimicking a structure similar to a TAD. In particular, this setting should favor 2D-aware tools, e.g. tools that exploit the spatial autocorrelation of the 2D Hi-C matrix (**FIND**, **Selfish**, and **sslHiC**).

Finally, the four matrices (from two conditions) were processed as described in “Type-I error control (H$_{0}$ setting)”, distinguishing results for the target zone from the others. The precision-recall (PR) curves based on adjusted $p$-value filtering were then obtained to simultaneously assess the precision (i.e. the ratio of bin pairs in the target zone among bin pairs declared positive) and the recall (i.e. the ratio of bin pairs declared positive among bin pairs in the target zone). Note that the recall is also named power in the framework of statistical tests and that “$1-$ Precision” indicates if the test properly controls the FDR.

Table 3 gives the total number of bin pairs for each chromosome and resolution, as well as the number of bin pairs in the target zone.

**Table 3:** Total number of bin pairs (third column) and number of bin pairs in the target zone for the evaluation of true positive detection rates (fourth column; H$_{1}$ setting only), for each chromosome and resolution

Chr.	Resolution	Total	In target
1	1 Mb	26 741	1275
7	1 Mb	12 765	861
21	1 Mb	861	45
1	500 kb	105 231	5151
7	500 kb	49 967	1888
21	500 kb	3218	136
1	200 kb	637 599	31 375
7	200 kb	306 209	10 936
21	200 kb	17 558	730

Note that, due to filters in the tools, not all these bin pairs were actually tested for each method.

### CTCF depletion study

To test the tools on a real life use case, we retrieved publicly available data from a CTCF depletion study in post-mitotic mouse cells [59]. This study features a Hi-C chromatin structure profiling of a murine erythroblast cell line under two conditions: either under accute depletion of CTCF through an auxin-inducible degron system (CTCF- condition) or in the control condition without auxin-induced depletion (CTCF+ condition). Hi-C libraries were generated, sequenced, and processed for three biological replicates per condition. We downloaded the six corresponding interaction matrices (GEO Accession GSE168251) and ran all the tools on each autosome independently at the resolution of 100 kb.

Although no precise and exhaustive ground truth exists for such a real case dataset, it is well known that the CTCF protein plays a major role in chromatin loop and TAD boundary formation. As reported in the original study, many structural differences are therefore expected between the two conditions, involving in particular genomic regions with a high density of active CTCF binding sites [59]. In order to assess the biological consistency of the predicted differential interactions, we compared the corresponding genomic positions with those of the active CTCF binding sites that were profiled by ChIP-seq experiments on the same cell line (GEO ACCESSION GSE129997, [60]). More precisely, for each 100 kb bin of the genome, we both computed:

- the number of times this bin was included in a bin pair found significantly different by the tool;
- the number of CTCF active sites (called peaks) present in this bin pair.

The joint distribution of these two quantities was thus obtained, and the Spearman correlation was computed to assess the general biological consistency of each tool’s results.

### Computational time

All tools were tested on the same infrastructure (Genotoul-Bioinfo cluster) on a single CPU node, except for **sslHiC** that was tested on a different node because it required GPU. For comparison purposes, we ran the tools on one processor only and recorded computational times in the H$_{1}$ setting and for the CTCF depletion study.

Processed data as used in the numerical experiments along with scripts implementing the different tools and performing the result analysis are made available at https://forgemia.inra.fr/scales/replication-chrocodiff.

## Results

### Number of tested bin pairs

**Figure 3:**
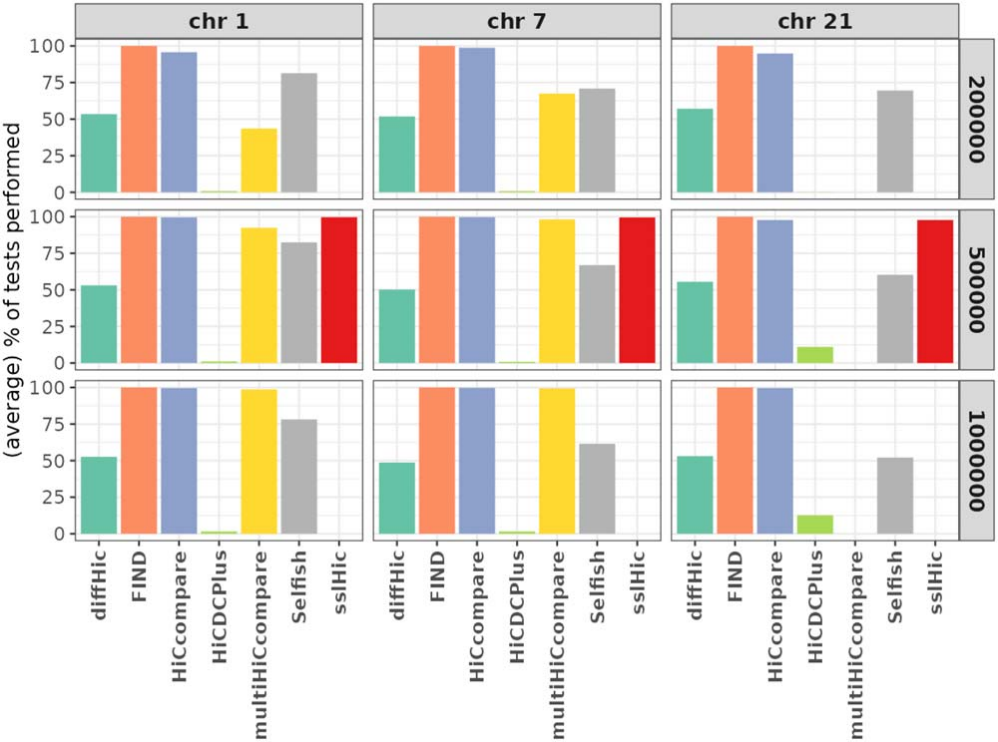
**Average percentage of performed tests** (across the 10 repeats) compared to the number of bin pairs passed as input to the tool (given in Table 3) across chromosomes and resolutions (200kb, 500kb, and 1Mb) in the H$_{0}$ setting. **sslHiC** could only be used on 500 kb resolution data.

We used the H$_{0}$ setting to assess the differences in the number of bin pairs filtered before the test procedure by the different tools. Figure 3 provides the proportion of tests performed for each tool in the H$_{0}$ setting (relative to the maximum number of possible tests, as given in Table 3 for each chromosome and resolution). The difference in the numbers of tested bins is thus only due to differences in the filtering step.

The different tools apply pre-filtering steps that resulted in a very different number of tested bin pairs. **HiCcompare** performed a number of tests that is constantly close to the maximum and **HiCDCPlus** constantly performed a very low number of tests because it only tests (the union of) regions with an interaction considered significantly above the interaction background (FDR adjusted $p$-value < 10%). For the relatively short chromosome 21, **multiHiCcompare** did not perform any test (all interactions were filtered out at preprocessing). However, it performed a number of tests close the maximum for the other two chromosomes at resolutions 500 kb and 1 Mb. At a 500 kb resolution (the only resolution available for this tool), **sslHiC** performed a number of tests close to the maximum for the three chromosomes. Finally, **diffHic** and **Selfish** filtered out approximately half of the bin pairs.

Note that the differences in the number of tested bin pairs are partially due to default values set differently by different tools for the same parameter: For instance, **HiCcompare** filters out bin pairs with an average $A$ value smaller than the 10th percentile of $A$ values while **multiHiCcompare** filters out bin pairs with an average $A$ value smaller than 5.

### Type-I error control (H$_{0}$ setting)

**Figure 4:**
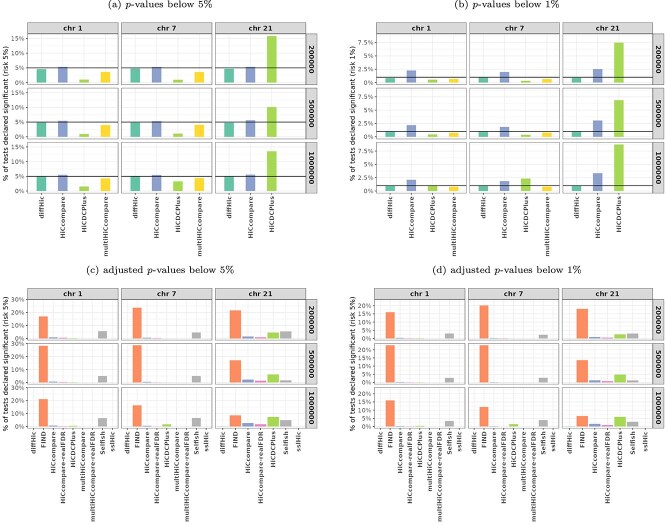
**Percentage of tests declared significant** (H$_{0}$ setting) for three chromosomes and three resolutions (200kb, 500kb, and 1Mb). Decisions are taken based on $p$-values (top) and adjusted $p$-values (bottom) with 5% (left) and 1% (right) risks. The black horizontal line (top figures) indicates the risk controlled by raw $p$-values. When the method did not perform a global FDR correction (see Table 1), we re-computed the adjusted $p$-values with the BH method applied to raw $p$-values (when available). This corresponds to columns named “*XXX*-realFDR” (bottom figures). **sslHiC** could only be used on 500 kb resolution data and **multiHiCcompare** performed no test on chromosome 21 because of its filtering step.

Figure 4 provides the percentage of tests declared significant for all chromosomes, resolutions, and tools, based on a 5% and a 1% thresholding of $p$-values and adjusted $p$-values. Figure S1 in Supplementary material additionally provides the same plots for a 10% threshold.

In H$_{0}$ settings, the percentage of $p$-values below 5% is expected to be at most 5% if the test is properly calibrated (type-I error control). A percentage much smaller than 5% indicates that the Type-I error control is valid but that the tool is conservative, suggesting that the test may be underpowered in non-H$_{0}$ situations. Also, since some tools only returned the adjusted $p$-value, we also gave the percentage of adjusted $p$-values below 5%. Since $p$-values are adjusted to control the FDR, this percentage is expected to be 0 if the test is properly calibrated. However, it is not possible to assess how conservative the test is only based on adjusted $p$-values.

The results shown in Fig. 4 are remarkably consistent across resolutions. This illustrates the fact that since FDR corresponds to a proportion of false positives, FDR control is *a priori* designed to be comparable across studies with different numbers of tests. Overall, the results show that only **diffHic** and **multiHiCcompare** properly controlled the Type-I error on this dataset, with a percentage of tests declared positive very close to the expected value. Nonetheless, for chromosome 21, **multiHiCcompare** did not perform any test as discussed above (see Fig. 3).

**HiCcompare**, which does not account for replicates and hence for variability within conditions, suffered from a small excess of false positives (e.g. chromosome 21, 1 Mb resolution, 1% risk). On the contrary, **HiCDCPlus** detected very few false positive results, except for chromosome 21 which displays a massive excess of false positives. This discrepancy between chromosomes could be related to the very low proportion of bin pairs passing the **HiCDCPlus** filters (see Fig. 3). Both **Selfish** and, in particular, **FIND** produced a large number of false positives, as visible in the plots based on adjusted $p$-values (bottom). For both methods, this could be explained by a statistical issue in the definition of the bin pair-level $p$-value (lack of multiple testing correction across radii for **Selfish**, and incorrect assumption of independence between aggregated $p$-values for **FIND**), as explained in Section “Methodological background of the tools.” **sslHiC** did not return any positive result based on adjusted $p$-value thresholding (which is the expected behavior). However, since it does not provide raw $p$-values, we were not able to assess its proper control of Type-I error. Finally, no large difference was observed between the standard BH correction (“*XXX*-realFDR”) and the multiple test correction implemented in **multiHiCcompare** and **HiCcompare**. This is confirmed by the strong linear relationship between these two quantities (Supplementary Fig. S2).

**Figure 5:**
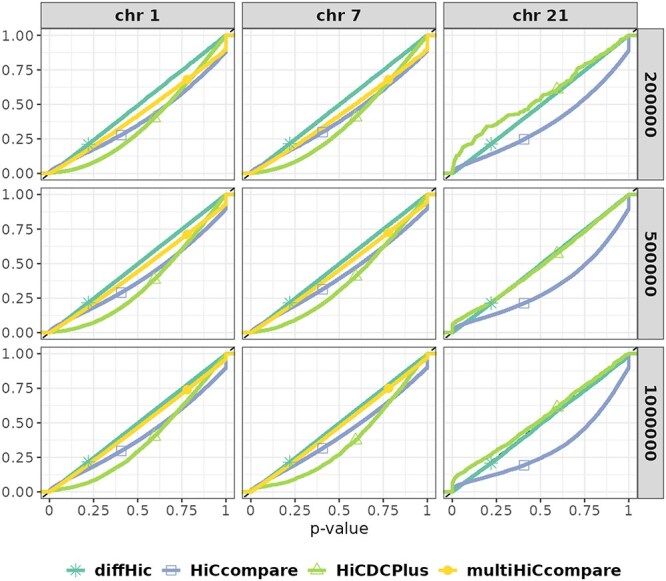
**ECDF of $p$-values** (H$_{0}$ setting). Well-calibrated tools are expected to have an ECDF that closely follows the diagonal, corresponding to a uniform distribution of $p$-values under H$_{0}$. An ECDF below the diagonal indicates a valid but conservative test, while an ECDF above the diagonal indicates that the test is not properly calibrated, yielding an excess of false positives. **multiHiCcompare** performed no test on chromosome 21 because of its filtering step.

For the tools that returned unadjusted $p$-values, Fig. 5 provides the ECDF of $p$-values. Note that the data displayed in Fig. 4 correspond to the values of the ECDF at risk $x=5$% (a) and 1% (b), respectively.

For all resolutions and chromosomes, **diffHic** was the tool closest to the expected uniform distribution, followed closely by the slightly conservative **multiHiCcompare** (Fig. 5). **HiCcompare** exhibited a slight excess of very small $p$-values; in the area where the $p$-value is below 0.1%, the ECDF of **HiCcompare** was frequently above the diagonal (see also Fig. 4). This behavior can be explained by its incapacity to account for variability across replicates of one condition, resulting in an excess of false positives. In contrast, **HiCDCPlus** generally displayed the opposite behavior, suggesting a lack of power (especially for chromosomes 1 and 7). However, it occasionally presented a strong excess of false positives, as observed on chromosome 21.

### Precision and Recall (H$_{1}$ setting)

**Figure 6:**
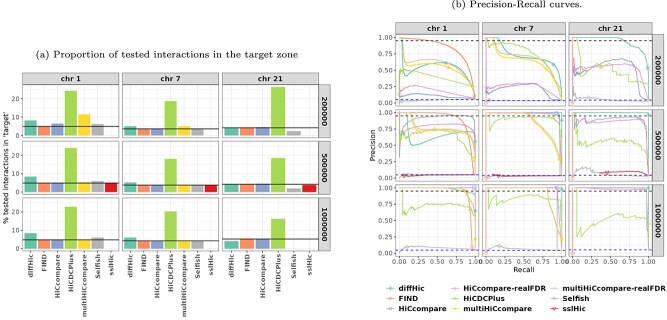
**Results for H$_{1}$ setting**. (a) Proportion of tested interactions that are in the target zone for each tool. The horizontal line indicates the proportion of interactions within the target zone of the original data before filtering. The reported proportions ($y$-axis) lie above or below the horizontal line depending on whether the tools ($x$-axis) predominantly filter interactions outside or inside the target zone, respectively. (b) PR curves computed from adjusted $p$-values, displaying recall (or power, $x$-axis) and precision ($y$-axis). For each method, the point corresponding to a threshold $=0.05$ (target FDR, as claimed by the method) is marked with a specific symbol. For methods that filtered out all interactions in the target zone before the test, the Recall cannot be computed (because the denominator would be 0). In this case, we arbitrarily represented them with a circle at $(0,0)$. Finally, the top horizontal dashed line (in black) corresponds to a Precision of 95% and the bottom horizontal dashed line (in blue) corresponds to the precision expectation of a uniform random draw of interactions. **sslHiC** could only be used on 500 kb resolution data and **multiHiCcompare** performed no test on chromosome 21 because of its filtering step.

Figure 6a provides the proportion of tested interactions that are located within the target zone, where positive calls are expected (true signal). This proportion may vary even for tools that rely on similar models or methods, because different data filtering methods are applied before testing for differential interaction. Results confirmed that this filtering step can have *per se* a strong impact on the test. In particular, **HiCDCPlus** predominantly discarded interactions outside the target zone rather than inside, which may be a desirable behavior. However, the overall number of retained interactions was generally very low for this tool (see Fig. 3 and the corresponding discussion). The other tools tended to generally have a proportion of tested interactions in the target zone close to the corresponding proportion in the original matrix (before filtering).

Figure 6b displays PR curves based on predictions computed from adjusted $p$-values, for all chromosomes, resolutions, and tools. An ideal classifier would have a precision of one and a recall of one. The resulting PR curve, based on various adjusted $p$-value thresholds, would then be the horizontal line joining the point at $(0,1)$ coordinates to point at $(1,1)$ coordinates (i.e. varying power depending on the number of interactions selected by the threshold, but all true positives), followed by a vertical line joining $(1,1)$ and $(1,0)$ (i.e. negative interactions are all selected after the positive one, for larger thresholds). The symbols on the PR curves in Fig. 6 b indicate for each tool the obtained precision and recall when thresholding the adjusted $p$-values provided at risk 5%, a threshold that corresponds to standard practice. A well calibrated tool should have a precision above the dashed horizontal line at 95% (for a clearer visualization of these results, Fig. S3 in Supplementary material also provides the obtained precision and recall for additional adjusted $p$-values thresholds).

In Fig. 6b, **diffHic** appears to be one of the best tools in the H$_{1}$ setting, as it yields curves closest to the ideal classifier in a majority of cases. In particular, it performed best on smaller chromosomes and at higher resolutions. This variability seems to be directly related to the number of performed tests: The smaller the number of tests (chromosome 21 or lower resolutions correspond to smaller numbers of interactions), the better the performance. However, in a number of cases, it did not properly control the FDR (the symbol corresponding to the 5% threshold of $p$-values is below the vertical dotted line at 95%). For instance, for chromosome 1, resolution 200 kb, the precision of **diffHic** was between 50 and 75% for the three thresholds.

**multiHiCcompare** and **HiCcompare** often displayed similar performances (and sometimes better than **diffHic**), with a marked disadvantage of **HiCcompare** for the highest resolution (200 kb). Aside from this resolution, and despite not utilizing information on biological replicates, **HiCcompare** achieved slightly better results than **multiHiCcompare** overall. Note that, similarly to the H$_{0}$ setting, the difference between the standard BH correction (“*XXX*-realFDR”) and the multiple testing correction implemented in **multiHiCcompare** and **HiCcompare** is small, with a slight improvement of performances when the standard BH correction is used. However, none of the two tools and the two versions of the correction properly controls the FDR at 5%. The only exceptions are **HiCcompare** at resolutions 1 Mb and 500 kb, but only for chromosomes 1 and 7 and with a recall of zero for chromosome 1.

From a PR curve point of view, **FIND** performed rather well for chromosome $1$, especially at resolutions $500$ kb and $1$ Mb with a curve consistently close or above the ideal classifier. However, its performances were bad for chromosomes $7$ and $21$, as all interactions had an adjusted $p$-value equal to 1. Note that, even for chromosome 1, **FIND** was far from properly controlling the FDR when thresholding the adjusted $p$-value. In all cases, its precision was close to 0.

**HiCDCPlus** had a rather heterogeneous and mild performance across chromosomes and resolutions. However, it was systematically the second or third best performing method.

Finally, although being somewhat heterogeneous between chromosomes and resolutions, **Selfish** and **sslHiC** had poor performances on this benchmark, altogether with PR curves usually close or below those of a random classifier.

### CTCF depletion study

In order to test the tools in a realistic setting on a full size dataset, we retrieved and analyzed genome-wide Hi-C matrices from a CTCF depletion study in mouse [59] (see Methods).

**Figure 7:**
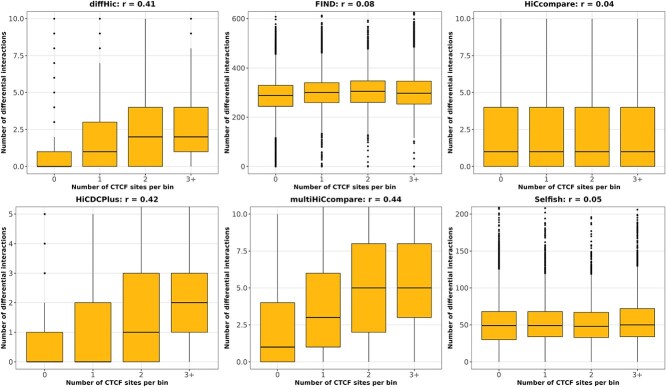
**Joint distribution of the number of CTCF sites and the number of differential interactions** per genomic bin of 100 kb in the CTCF depletion dataset. Each boxplot represents the distribution of differential interactions ($y$-axis) obtained by a given tool (one per panel) between matrices from the CTCF+ and the CTCF- conditions for genomic bins with 0, 1, 2, or at least 3 CTCF sites ($x$-axis). The Spearman correlation between these values across all bins is provided for each tool ($r$). Bins with many CTCF sites are expected to be predominantly involved in differential interactions upon CTCF depletion compared to bins with few CTCF sites, as observed in results from **diffHic** and **HiCDCPlus** for instance (left side).

Figure 7 provides for each tool the joint distribution over the 100 kb bins of the genome between the number of CTCF sites present in the bin ($x$-axis) and the number of differential interactions in which the bin was involved after comparing matrices from the CTCF+ and the CTCF- conditions ($y$-axis). Since CTCF depletion is expected to predominantly impact genomic regions with CTCF binding sites, a positive correlation should be observed between these quantities.

This was the case for some of the tools. In particular, Spearman’s correlation values of $r = 0.44$, $r = 0.42$, and $r = 0.41$ were, respectively, obtained for **multiHiCcompare**, **HiCDCPlus,** and **diffHic**. Globally, these tools detected more differential interactions between CTCF-rich regions than between CTCF-poor regions, as expected. However, in line with previous results from the H$_{0}$ setting (Fig. 3), **HiCDCPlus** realized a low number of tests compared to the total number of bin pairs in the whole dataset. Less than 5% of bin pairs were kept after the filtering step (Supplementary Fig. S4).

On the contrary, no substantial correlation was obtained for the other tools, with $r=0.04$ for **HiCcompare**, $r= 0.05$ for **Selfish**, and $r= 0.08$ for **FIND**.

### Computational time

**Figure 8:**
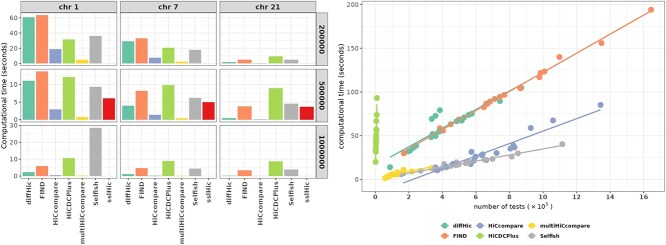
**Computational time.** Left: Computational time in seconds ($y$-axis) needed for each tool ($y$-axis) to run in the H$_{1}$ setting. **sslHiC** could only be used on 500 kb resolution data and **multiHiCcompare** performed no test on chromosome 21 because of its filtering step. Right: Computational time in seconds ($y$-axis) versus the number of tests performed in a given chromosome ($y$-axis) for the CTCF depletion dataset. **sslHiC** could not be used.

Figure 8 shows the computational time required for performing the tests in the H$_{1}$ setting and for the CTCF depletion dataset. In addition, Supplementary Fig. S5 provides another representation of these results for the H$_{1}$ setting with respect to the number of tests performed, and Supplementary Fig. S6 gives the total computational time required for the CTCF depletion dataset.

**FIND** exhibited large computational times for certain chromosomes and resolutions. However, the scalability of this tool was not the worst: **HiCDCPlus** showed the greatest increase in computational time with respect to the number of performed tests. This can primarily be attributed to the fact that its filtering steps removed most bin pairs, and its computational time scaled with the total number of bin pairs rather than the number of bin pairs remaining after filtering. The tools showing the best scalability were **HiCcompare**, **multiHiCcompare**, and **Selfish**. This result may be partly attributed to the faster implementation of the cyclic LOESS (used for MA and MD normalization) available in **HiCcompare** and **multiHiCcompare**, but not in **diffHic**.

Finally, as **sslHiC** is designed to run on GPU processors, its computational time could not be directly compared with that of the other tools. Despite its relatively short runtime, its computational resource requirements were substantial.

## Discussion and conclusion

Our benchmark allowed to evaluate and compare the statistical performances of Hi-C data differential analysis tools on practical examples. Importantly, the results revealed that the FDR was not properly controlled across all tools. This could be due to the small number of samples in our experiments (only two per condition for the H$_{1}$ setting), highlighting the importance of that factor. Nonetheless, some tools—particularly **diffHic**—still managed to correctly control the Type-I error rate in the H$_{0}$ setting. Additionally, the per-distance-basis FDR correction appeared to have a limited effect, especially when applied to a single chromosome.

Globally, in our benchmark, **diffHic** delivered the best results. It properly controlled the Type-I error rate in the H$_{0}$ setting and was the only tool to properly control the FDR in some cases. Its power, for a 5% risk, was also among the best, always larger than 50% and generally close to 100%. Interestingly, for the lowest resolution, **HiCcompare** also showed interesting performances in the H$_{1}$ setting but always exhibited an inflated number of false positives in the H$_{0}$ setting and gave disappointing results in the CTCF depletion use case.

**diffHic** and **multiHiCcompare** produced comparable results in the H$_{0}$ setting and, in terms of PR curves, in the H$_{1}$ setting. Both tools also showed good biological consistency between Hi-C and ChIP-seq data in the CTCF depletion analysis. This alignment was expected, as they rely on the same model. However, the performance differences observed between the two tools underscore the importance of filtering and the choice of default parameters. Notably, the default filters in **multiHiCcompare** seemed sometimes too stringent (e.g. no results were obtained for some chromosomes in both the simulated and real-world experiments). Additionally, the FPR of **multiHiCcompare** consistently exceeded 30% (and often surpassed 50%) for a 5% risk threshold. The strong impact of preprocessing steps is unsurprising and has been previously acknowledged in other omics studies [61].

**HiCDCPlus** was also found to be overly stringent in its filtering step, consistently performing a very low number of tests. However, in the CTCF depletion application, it produced good results.

**FIND** presents an interesting case. In both simulation settings, the tool tended to predict too many false positives for a given threshold. However, on chromosome 1 in the H$_{1}$ setting, it demonstrated excellent ordering of interactions based on adjusted $p$-values, with the PR curve closely approaching that of a perfect classifier. This suggests that the adjusted $p$-values returned by **FIND** can serve as a reliable score for ranking interactions by significance level, although they cannot be statistically interpreted. In this case, using higher thresholds than typically expected is recommended. However, for chromosomes 7 and 21, all adjusted $p$-values returned by **FIND** were equal to 1. Overall, contrary to **diffHic**, the performance differences observed with **FIND** do not seem to be directly related to the number of tested interactions. For instance, in the H$_{1}$ setting, **FIND** performed better on the largest chromosome but worse for the highest resolution of 200 kb, which has more interactions to test.

Interestingly, 2D-aware tools such as **FIND**, **Selfish**, and **sslHiC** leverage the spatial auto-correlation inherent to the 2D Hi-C matrices in their modeling. However, these tools did not generally show superior performances, even in situations like the H$_{1}$ setting, where there is a strong spatial dependency in differential interaction locations within the 2D Hi-C matrix. This suggests that the current methods for incorporating spatial 2D structure may not be effectively capturing its relevance.

Finally, it is worth noting that the currently available tools are still unable to accommodate a wide variety of study designs. Few methods allow to use covariates (see Table 1) and, to the best of our knowledge, no tool is capable of properly handling paired data (e.g. differences between two tissues, with multiple individuals each providing a pair of tissue samples as replicates) or repeated measurement designs (similarly to what is done in mixed models).

In this study, we focused on two datasets, encompassing two simulation settings and a real-world application. Expanding the investigation to include broader datasets and experimental settings would be valuable to assess the robustness of our conclusions across more varied designs, resolutions, and size effects. Additionally, our analysis underscored the need for a deeper understanding of the complex interplay between preprocessing steps—particularly normalization types and filtering—and the models used.

**Key Points**

- We reviewed and benchmarked available tools for differential analysis of Hi-C matrices.
- Preprocessing steps differed between tools, strongly impacting the results, even for tools with the same type of model.
- None of the tools properly controlled the FDR at the expected rate in our simulation setting. However, some tools effectively controlled the Type-I error in situations where no signal was expected in the data.
- In our simulations, **diffHic** yielded the best overall results. Currently, tools based on a 2D-aware model did not outperform the others.
- Our review highlighted the need for models and tools able to handle paired designs and repeated measurement designs.

## Supplementary Material

jorge_etal_p2024-suppmat_bbaf074

## Data Availability

For the H$_{0}$ and H$_{1}$ settings, raw sequencing data were obtained from the ENCODE project https://www.encodeproject.org/ using accession ENCSR295BDK. Processed Hi-C data (by chromosome, resolution, and technical replicates) and corresponding quality controls are available at https://doi.org/10.57745/LR0W9R. For the CTCF dataset, Hi-C matrices and ChIP-seq peaks were retrieved from GEO using accession GSE168251 and GSE129997, respectively. Processed Hi-C data (by chromosome at 100 kb resolution) and converter script are available at https://doi.org/10.57745/LR0W9R.
