# Supplementary material for: A comprehensive review and benchmark of differential analysis tools for Hi-C data
Source: Brief Bioinform. 2025 Mar 4;26(2):bbaf074. doi: 10.1093/bib/bbaf074 (PMC11879411; doi:10.1093/bib/bbaf074)
Supplement: jorge_etal_p2024-suppmat_bbaf074 [file jorge_etal_p2024-suppmat_bbaf074.pdf]

Supplementary information for “A comprehensive review and benchmark of differential analysis tools for Hi-C data”

Élise Jorge, Sylvain Foissac, Pierre Neuvial, Matthias Zytnicki, and Nathalie Vialaneix

Contents

1 Supplementary tables 1

2 Supplementary figures 2

3 Supplementary information on documentation and installation of tools 6
- 3.1 Documentation . . . 6
- 3.2 Bugs identified during tool installation . . . 6

4 Supplementary methods 7
- 4.1 Hi-C data processing of ENCODE human data . . . 7
- 4.2 Hi-C data processing of CTCF data . . . 7

1 Supplementary tables

Tool	Filter type	Mandatory?
**ACCOST**	mappability threshold	yes (but can be bypassed using a threshold of 0 or by setting mappability to 1)
**CHESS**	mappability threshold	no
**diffHic**	total count user-specified threshold<br>total count data-driven threshold at matrix level<br>total count data-driven threshold at bin distance level<br>remove diagonal bin pairs<br>select only peaks	no<br>no<br>no<br>no<br>no
**HiCcompare**	user-specified	no
**HiCDCPlus**	filters out bin pairs with a non significant effect according to distance, GC, and mappability	yes (but not all effects are mandatory to consider)
**HOMER**	total count data-driven threshold at matrix level	no
**multiHiCcompare**	total count user-specified threshold	no
**Selfish**	total count user-specified threshold<br>remove bin pairs for which genomic distance is above a certain threshold	no<br>no

Table S1: Types of filters available in the different tools.

Tool	Normalization type	Performed with?	Mandatory?
**ACCOST**	normalization of total counts between bins	user-provided bin-specific correction values	yes
	genomic distance effect correction	data-driven distance-specific correction value	yes
**CHESS**	genomic distance effect correction	observed over expected	yes
**diffHic**	normalization of matrix sequencing depth	MA correction	no (but highly recommended)
	normalization of total counts between bins	ICE	no
	other	CNV correction	no
**FIND**	normalization of total counts between bins	VC, VC_SQRT, KR	no
**HiCcompare**	normalization of matrix sequencing depth	MD correction	no (but highly recommended)
**HiCDCPlus**	genomic distance effect correction	data-driven distance-specific correction value	yes
**HOMER**	normalization of total counts between bins	ICE	yes
**multiHiCcompare**	normalization of matrix sequencing depth	MD correction	no (but highly recommended)
**Selfish**	normalization of total counts between bins	user-provided bin-specific correction values	no
	normalization of total counts between bins	KR	no
**sslHiC**	normalization of matrix sequencing depth	TSS	yes
	normalization of matrix sequencing depth	min/max normalization on $\log_{10}$-transformation	yes

Table S2: Types of normalization performed by the different tools.

Tool	Reference	Tool URL	Used version
**diffHic**	[5]	`https://www.bioconductor.org/packages/release/bioc/html/diffHic.html`	1.26.0
**FIND**	[2]	`https://bitbucket.org/nadhir/find`	1.0.0
**HiCcompare**	[10]	`https://www.bioconductor.org/packages/release/bioc/html/HiCcompare.html`	1.16.0
**HiCDCPlus**	[8]	`https://bioconductor.org/packages/release/bioc/html/HiCDCPlus.html`	1.2.1
**multiHiCcompare**	[9]	`https://www.bioconductor.org/packages/release/bioc/html/multiHiCcompare.html`	1.12.0
**Selfish**	[1]	`https://github.com/ay-lab/selfish`	as of 2022/09/01
**sslHic**	[4]	`https://github.com/lihan97/sslHiC`	as of 2023/04/11

Table S3: Tested tools, reference, source and number of the used version.

2 Supplementary figures

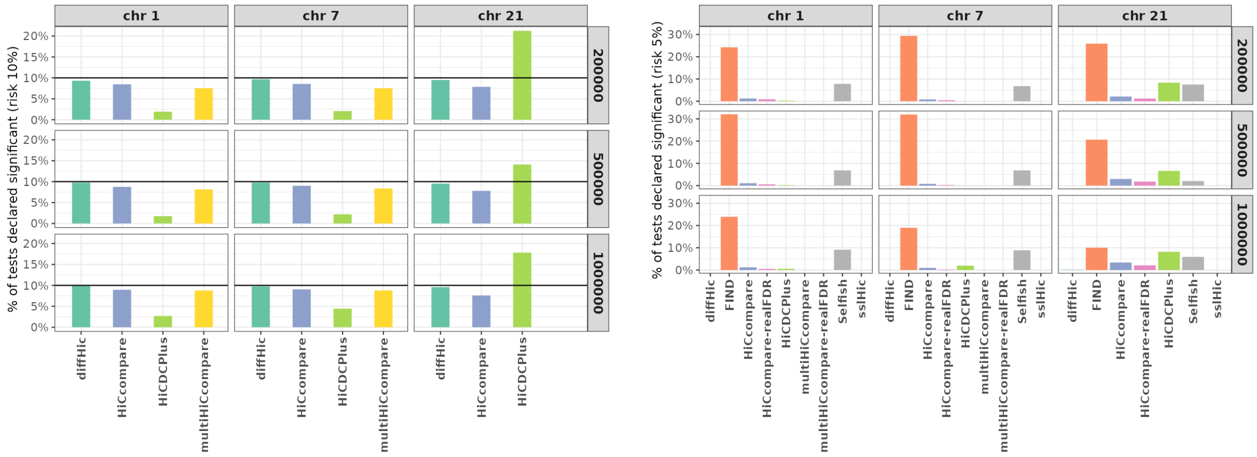

Figure S1: **"H_0 setting".** Percentage of tests declared significant at risk 10% based on p-values (left) and adjusted p-values (right). The black horizontal line (left) indicates the risk level that raw p-values are supposed to control. **sslHiC** could only be used on 500 kb resolution data and **multiHiCcompare** performed no test on chromosome 21 because of its filtering step.

Tool	Option name	Description	Default value	Used value
**diffHic**	Default values were used. We did not perform filtering based on `aveLogCPM` but performed the trended filter as recommended in the described analysis pipeline.			
**FIND**	`qvalue`	q-value threshold for exported results	0.001	1
	`chunkSize`	no documentation but used to split the computation into small chunks	50	8
	`nbProcessor`	no documentation but used for multicore processing	2	1
**HiCcompare**	Default values were used.			
**HiCDCPlus**	Default values were used.			
**multiHiCcompare**	Default values were used.			
**selfish**	Default values were used.			
**sslHiC**	Default values were used.			

Table S4: Options set to run the different tools. For all other parameters, we used the default values.

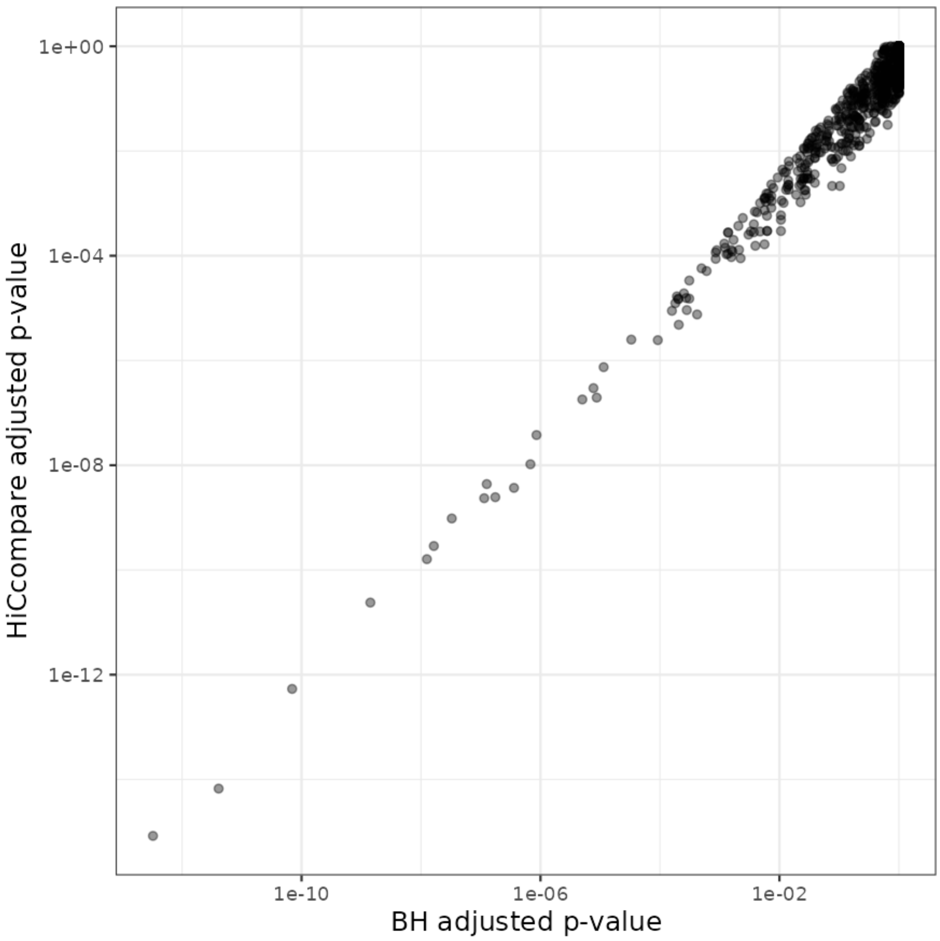

Figure S2: **"$\mathbf{H_0}$ setting".** Adjusted p-values as implemented in the R package **HiCcompare** versus standard BH adjusted p-values. This plot corresponds to results obtained on chromosome 21 at 1 Mb resolution and axes have been $\log_{10}$-scaled.

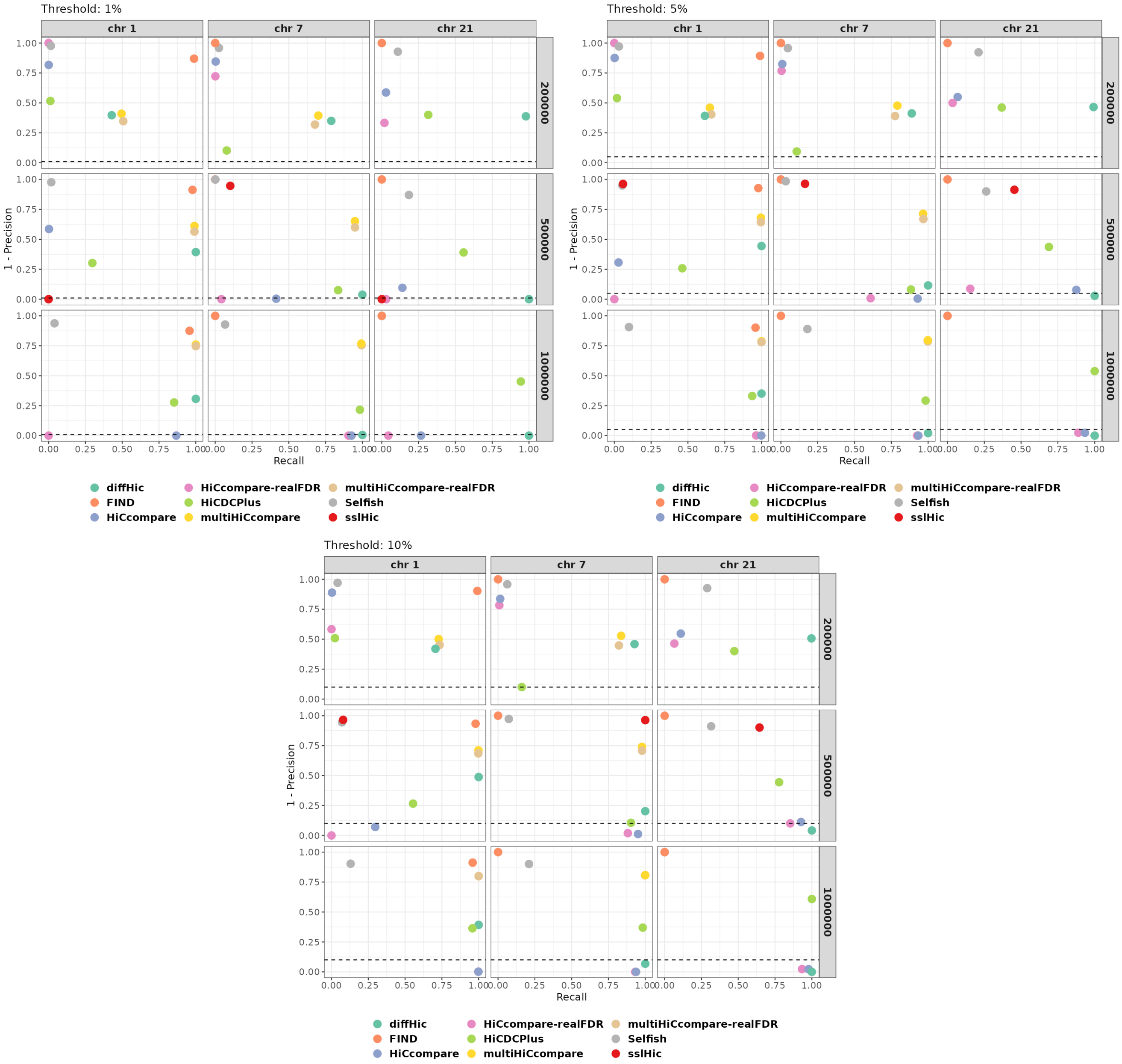

Figure S3: **"H_1 setting".** 1− Precision versus Recall at different thresholds of the adjusted p-values (1%, 5%, and 10%). The dashed horizontal line shows the target FDR that the test should control. **sslHiC** could only be used on 500 kb resolution data and **multiHiCcompare** performed no test on chromosome 21 because of its filtering step.

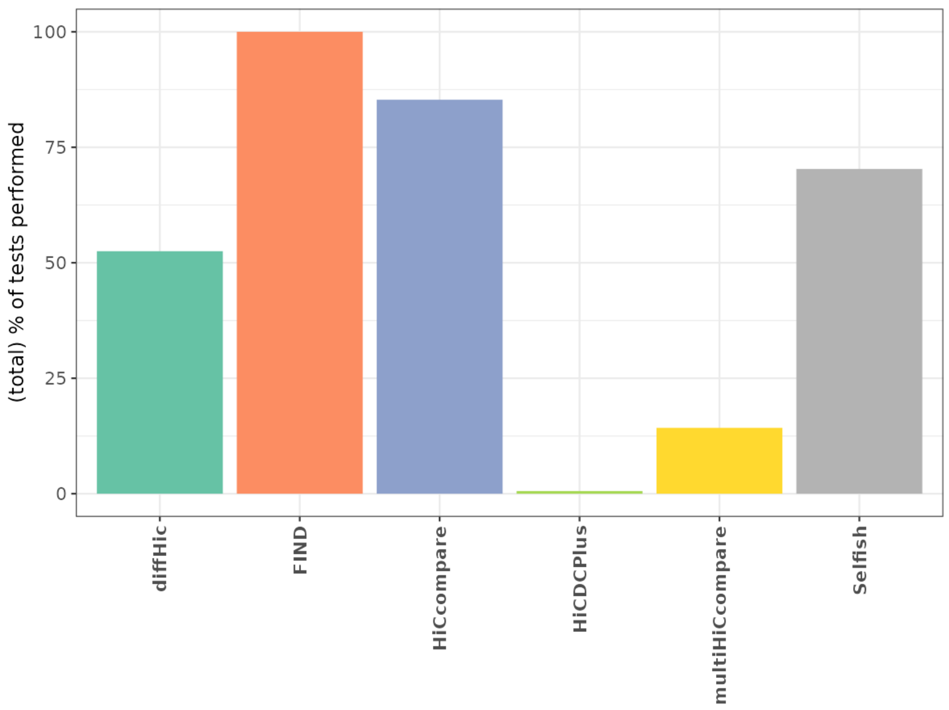

Figure S4: **CTCF depletion dataset.** Percentage of tests performed by each tool (after filtering steps) compared to the original number of bin pairs available in data. **sslHiC** could not be used.

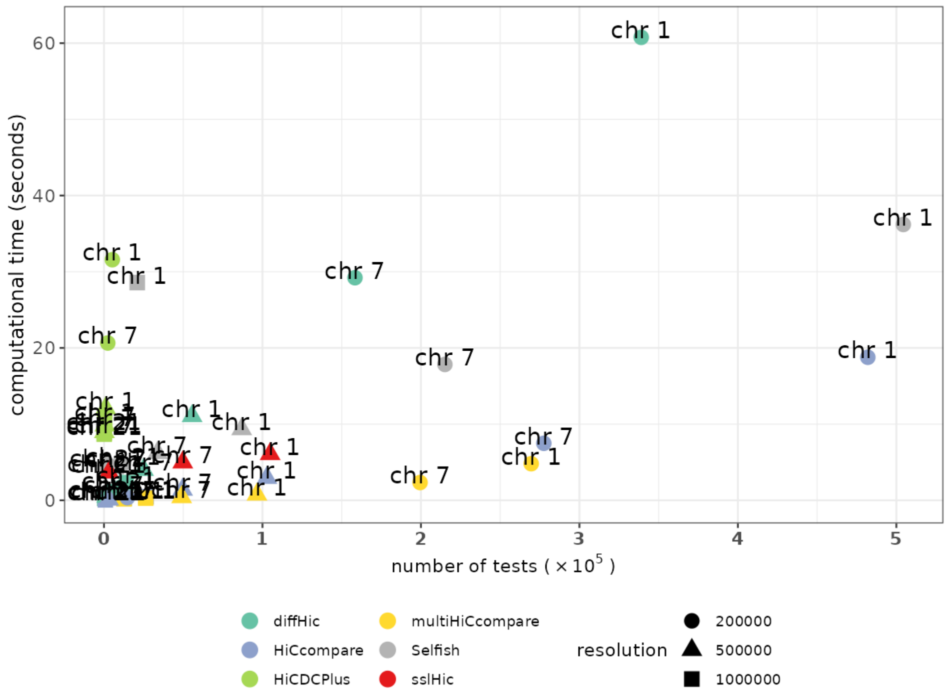

Figure S5: **"H_1 setting".** Computational time with respect to performed number of tests (remaining after the filtering step). **FIND** computational time is not provided because it was too large compare to the other tools to not compromise the plot readability. **sslHiC** could only be used on 500 kb resolution data and **multiHiCcompare** performed no test on chromosome 21 because of its filtering step.

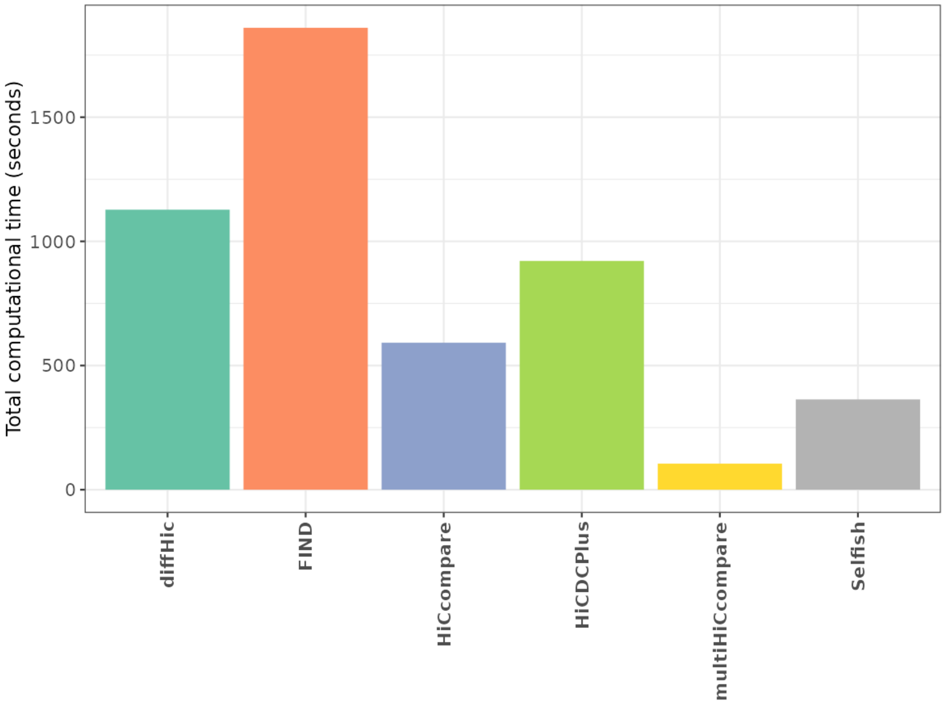

Figure S6: **CTCF depletion dataset.** Total computational time required by each tool. **sslHiC** could not be used.

3 Supplementary information on documentation and installation of tools

3.1 Documentation

The documentation is of varying precision: Bioconductor R packages (**diffHic**, **HiCcompare**, **HiCDCPlus**, and **multiHiCcompare**) are particularly easy to use thanks to their extensive documentation. On the contrary, **FIND** documentation is incomplete and imprecise: For instance, only some of the exported functions are documented (*e.g.*, among the methods to extract differential analysis results, `getDCIs_XXX`, only the one for matrix lists is documented whereas the method designed for the outputs of the import function `loadHicExperiment` is not documented), the documentation of some arguments is not clear (*e.g.*, "qvalue: numeric. cutoff q-value. Taken into considiration i", in which the sentence is cut midway), and the documentation of some other arguments is missing (*e.g.*, `isrOP_qval`). In addition, **FIND** includes a vignette but this vignette is not automatically built during the installation (it has dependencies to packages that are not listed in the "required packages" section of the documentation). When we tried to build it with **knitr** after installing the tool, it yielded to a segmentation fault of undefined origin.

Selfish and **sslHiC** offer a basic documentation in the `README` file of their code repository, with a description of all the function parameters and a basic example. **ACCOST** has approximately the same level of documentation, included in a HTML manual in their code repository (note that this manual can not be read online and has to be downloaded to be used). In contrast, **CHESS** provides a complete "Read the Docs" documentation at `https://chess-hic.readthedocs.io`, with installation instruction, examples, parameter description, and some practical advices on how to set these parameters with your own data.

Finally, **HOMER** is a set of Perl scripts, with some dependencies to external R packages (as **edgeR**, [7]). The installation instructions are detailed though not easy to follow for a basic user (Windows users are instructed to emulate a linux environment to use **HOMER**). An installation script is provided to download a ZIP file with the source code (that does not seem to be available in a standard code repository). The documentation is also provided on the tool website `http://homer.ucsd.edu/homer/`. It is detailed but not easy to navigate and finding the documentation related to differential analysis `http://homer.ucsd.edu/homer/interactions/HiCinteractions.html` might require some efforts.

3.2 Bugs identified during tool installation

Some tools could not be installed on our infrastructure just following the installation instructions, and some of the problems most likely were caused by a bug in the tool. When we thought we found a bug, we filed an issue to the code repository:

- sslHiC
  - `https://github.com/lihan97/sslHiC/issues/2`
  - `https://github.com/lihan97/sslHiC/issues/3`

- **selfish**
  - https://github.com/ay-lab/selfish/issues/26

HOMER does not include an issue tracker and an email was used instead.

4 Supplementary methods

4.1 Hi-C data processing of ENCODE human data

To perform the simulations for the H_0 and H_1 settings, we used a human Hi-C dataset generated by the ENCODE consortium [11]. Raw reads from five sequencing runs of the same Hi-C library were downloaded in fastq format from the ENCODE data portal [6] at `https://www.encodeproject.org/` using the experiment accession ID ENCSR295BDK. This library comes from a colon sample of a 37-year old male donor, each sequencing run providing a technical replicate.

To obtain a Hi-C matrix for each replicate, raw sequencing reads were processed using the `nf-core/hic` pipeline [3] v1.2.2 on the assembly version GRCh38 of the human genome using the following arguments: `--min_mapq 10 --restriction_site '^GATC' --ligation_site 'GATCGATC' --min_insert_size 20 --max_insert_size 1000 --rm_singleton --rm_dup --skip_ice --bwt2_opts_end2end '--very-sensitive -L 30 --score-min L,-0.6,-0.2 --end-to-end --reorder' --bwt2_opts_trimmed '--very-sensitive -L 20 --score-min L,-0.6,-0.2 --end-to-end --reorder'`. Matrix visualizations were generated using the `hicPlotMatrix` function of `HiCExplorer` v3.7.2 with the parameters `--clearMaskedBins --log1 --colorMap OrRd --perChromosome --dpi 300 --fontsize 16 --rotationX 45`.

4.2 Hi-C data processing of CTCF data

To assess the biological relevance of the differential analysis results we used a large scale dataset from an existing study on the role of CTCF during cell cycle progression in mouse [13]. This study features an erythroblasts cell line that has been modified to integrate an auxin-induced depletion system that targets the CTCF protein concentration. In particular, Hi-C has been performed for three replicates of two conditions: a wild-type condition, with no CTCF depletion ("CTCF+"), and a CTCF depleted condition ("CTCF-"), both at the post-mitotic G1 phase, 120 minutes after cell cycle reentry. Raw interaction counts of the six Hi-C matrices were directly downloaded from the GEO platform uwing the accession ID "GSE168251" from `https://www.ncbi.nlm.nih.gov/geo/query/acc.cgi?acc=GSE168251`. File list: `GSM5133388_120min_no_a_rep1.cool.gz`, `GSM5133389_120min_no_a_rep2.cool.gz`, `GSM5133390_120min_no_a_rep3.cool.gz`, `GSM5133391_120min_with_a_rep1.cool.gz`, `GSM5133392_120min_with_a_rep2.cool.gz`, and `GSM5133393_120min_with_a_rep3.cool.gz`. Matrices in cool format were exported using the HiC-Pro text format by chromosome at 100 kb resolution. Obtained files were used as input for differential analysis with each tested tools using the "CTCF+" vs. "CTCF-" contrast. Resulting differences in interaction values (adjusted p-value $< 5\%$) were kept for further validation.

In order to assess the biological relevance of the resulting differential interactions according to each tested tool, in the absence of ground truth, we used an existing dataset of genome-wide CTCF occupancy that was obtained by ChIP-Seq experiment on the same murine cell line [12]. Resulting CTCF binding sites were directly downloaded in BED format from the GEO website using the accession ID GSE129997 (url: `https://www.ncbi.nlm.nih.gov/geo/query/acc.cgi?acc=GSE129997`). File name: `GSE129997_CTCF_midG1.narrowPeak.gz`.

CTCF occupancy data from ChIP-seq experiments and differential analysis results from each tool were integrated by projecting them on the `mm9` version of the mouse genome at the 100 kb bin level resolution using the **bedtools intersect -c** command. Each genomic bin was assigned two values: the number of differential interactions that involved the bin and the number of detected CTCF binding sites. Spearman correlation between these numbers was then computed across all bins using the `cor()` R function with the `method = "spearman"` parameter.

References

[1] Abbas Roayaei Ardakany, Ferhat Ay, and Stefano Lonardi. Selfish: discovery of differential chromatin interactions via a self-similarity measure. *Bioinformatics*, 35(14):i145–i153, 2019.

[2] Mohamed Nadhir Djekidel, Yang Chen, and Michael Q. Zhang. FIND: difFerential chromatin INteractions Detection using a spatial Poisson process. *Genome Research*, 28:412–422, 2018.

[3] Philip A. Ewels, Alexander Peltzer, Sven Fillinger, Harshil Patel, Johannes Alneberg, Andreas Wilm, Maxime Ulysse Garcia, Paolo Di Tommaso, and Sven Nahnsen. The nf-core framework for community-curated bioinformatics pipelines. *Nature Biotechnology*, 38:276–278, 2020.

[4] Han Li, Xuan He, Lawrence Kurowski, Ruotian Zhang, Dan Zhao, and Jianyang Zeng. Improving comparative analyses of Hi-C data via contrastive self-supervised learning. *Briefings in Bioinformatics*, 24(4):bbad193, 2023.

[5] Aaron T.L. Lun and Gordon K. Smyth. diffHic: a Bioconductor package to detect differential genomic interactions in Hi-C data. *BMC Bioinformatics*, 16:258, 2015.

[6] Yunhai Luo, Benjamin C Hitz, Idan Gabdank, Jason A Hilton, Meenakshi S Kagda, Bonita Lam, Zachary Myers, Paul Sud, Jennifer Jou, Khine Lin, Ulugbek K. Baymuradov, Keenan Graham, Casey Litton, Stuart R. Miyasato, J. Seth Strattan, Otto Jolank, Jin-Wook Lee, Forrest Y. Tanaka, Philipp Adenekan, Emma O'Neill, and J. Michael Cherry. New developments on the Encyclopedia of DNA Elements (ENCODE) data portal. *Nucleic Acids Research*, 48(D1):D882–D889, 2020.

[7] Mark D. Robinson, David J. McCarthy, and Gordon K. Smyth. edgeR: a Bioconductor package for differential expression analysis of digital gene expression data. *Bioinformatics*, 26(1):139–140, 2010.

[8] Merve Sahin, Wilfred Wong, Yingqian Zhan, Kinsey Van Deynze, Richard Koche, and Christina S. Leslie. HiC-DC+ enables systematic 3D interaction calls and differential analysis for Hi-C and HiChIP. *Nature Communications*, 12(1):3366, 2021.

[9] John C. Stansfield, Kellen G. Cresswell, and Mikhail G. Dozmorov. multiHiCcompare: joint normalization and comparative analysis of complex Hi-C experiments. *Bioinformatics*, 35(17):2916–2923, 2019.

[10] John C. Stansfield, Kellen G. Cresswell, Vladimir I. Vladimirov, and Mikhail G. Dozmorov. HiCcompare: an R-package for joint normalization and comparison of HI-C datasets. *BMC Bioinformatics*, 19:279, 2018.

[11] The ENCODE Project Consortium. An integrated encyclopedia of DNA elements in the human genome. *Nature*, 489(7414):57–74, 2012.

[12] Haoyue Zhang, Daniel J. Emerson, Thomas G. Gilgenast, Katelyn R. Titus, Yemin Lan, Peng Huang, Di Zhang, Hongxin Wang, Cheryl A. Keller, Belinda Giardine, Ross C. Hardison, Jennifer E. Phillips-Cremins, and Gerd A. Blobel. Chromatin structure dynamics during the mitosis-to-G1 phase transition. *Nature*, 576(7785):158–162, 2019.

[13] Haoyue Zhang, Jessica Lam, Di Zhang, Yemin Lan, Marit W. Vermunt, Cheryl A. Keller, Belinda Giardine, Ross C. Hardison, and Gerd A. Blobel. CTCF and transcription influence chromatin structure re-configuration after mitosis. *Nature Communications*, 12:5157, 2021.
